# Supplementary material for: Extended DNA-binding interfaces beyond the canonical SAP domain contribute to the function of replication stress regulator SDE2 at DNA replication forks
Source: J Biol Chem. 2022 Jul 16;298(8):102268. doi: 10.1016/j.jbc.2022.102268 (PMC9399289; doi:10.1016/j.jbc.2022.102268)
Supplement: Supporting Information [file mmc1.pdf]

Supporting Information

Extended DNA binding interfaces beyond the canonical SAP domain contribute to the function of replication stress regulator SDE2 at DNA replication forks

Alexandra S. Weinheimer[1,6], YiTing Paung[2,6], Julie Rageul[3], Arafat Khan[3], Natalie Lo[3], Brian Ho[3], Michael Tong[3], Sébastien Alphonse[4], Markus A. Seeliger[3,5,*], Hyungjin Kim[3,5,*]

[1]Department of Biochemistry and Cell Biology, State University of New York at Stony Brook, Stony Brook, New York 11794, USA
[2]Department of Chemistry, State University of New York at Stony Brook, Stony Brook, New York 11794, USA
[3]Department of Pharmacological Sciences, State University of New York at Stony Brook, Stony Brook, New York 11794, USA
[4]Department of Chemistry and Biochemistry, The City College of New York, New York 10031, USA
[5]Stony Brook Cancer Center, Renaissance School of Medicine at Stony Brook University, Stony Brook, New York 11794, USA
[6]These authors contributed equally

* For correspondence: Hyungjin.kim@stonybrook.edu; Markus.seeliger@stonybrook.edu

Table S1. SDM Primers

SDE2 Δ395-427	TTCACCTCTGTTGCA TCTGTCAGAGGACTG
SDE2 Δ428-451 (S428*)	CAG CAA GAC TCT TCT aaG TCA GAG GAC TGG CA
SDE2 K444A	CCG GCT TTA TTT GCC gcG CCT TTG AAA GGG AA
SDE2 K444 K447A	TTT GCC GCG CCT TTG gcA GGG AAG AAA AAA GA
SDE2 G417A	GA CTG AAA TGT GGG GcC ACT CTG CAG GAG CG
SDE2 G417A L419E	AAA TGT GGG GCC ACT gaG CAG GAG CGG GCA GC
SDE2 G412A L413E G417A	AA CTG ATG GCC CTT GcA gaG AAA TGT GGG GCC AC
SDE2 C415A G417A T418A	GCC CTT GGA CTG AAA gcT GGG GCC GCT CTG CA
SDE2 A417S A418V	GGA CTG AAA GCT GGG tCC GtT CTG CAG GAG CGG GC
SF3A3 Δ253-285	TTCTCCTCCTGGGAG AGTACCAAAGGAAAG
SF3A3 Δ286-311	GCCCAGAGACTATTC GACACTGAAAGGAAC
SF3A3 Δ253-311	TTCTCCTCCTGGGAG GACACTGAAAGGAAC

Table S2. Chemicals & Reagents

Chemical/Reagent	Manufacturer	Cat. #
5-ethynyl-2´-deoxyuridine (EdU)	ThermoFisher Scientific	A10044
Accugel 29:1 (40%)	National Diagnostics	EC-852
Amicon Ultra Centrifugal Filter Devices, 3K	MilliporeSigma	UFC500308
Amicon Ultra Centrifugal Filter Devices, 30K	MilliporeSigma	UFC503008
Biotin azide	ThermoFisher Scientific	B10184
BL21(DE3) Competent E. coli	New England Biolabs	C2527H
Click-iT® Cell Reaction Buffer Kit	ThermoFisher Scientific	C10269
cOmplete, EDTA-free protease inhibitor cocktail	MilliporeSigma	11873580001
DL-Dithiothreitol (DTT)	MilliporeSigma	D0632
Doxycycline hyclate	MilliporeSigma	D9891
Duolink® In Situ Detection Reagents Red	MilliporeSigma	DUO92008
Duolink® In Situ PLA® Probe Anti-Rabbit PLUS	MilliporeSigma	DUO92002
Duolink® In Situ PLA® Probe Anti-Mouse MINUS	MilliporeSigma	DUO92004
FLAG M2 affinity gel	MilliporeSigma	A2220
Hen egg white lysozyme	MilliporeSigma	L4919
Hexadimethrine bromide (polybrene)	MilliporeSigma	H9268
HRV 3C protease (2 units/μL)	Pierce/ThermoFisher Scientific	88946
Isopropyl β-d-1-thiogalactopyranoside (IPTG)	MilliporeSigma	I6758
GeneJuice Transfection Reagent	MilliporeSigma	70967
Glutathione agarose	Pierce/ThermoFisher Scientific	16100
GSTrap FF Column (1 x 5 mL)	Cytiva/GE	17513101
L-glutathione, reduced (GSH)	MilliporeSigma	G4251
Phenylmethanesulfonyl fluoride (PMSF)	MilliporeSigma	P7626
PES Protein Concentrator, 30K	Pierce/ThermoFisher Scientific	88529
Puromycin	MilliporeSigma	P8833
RNAi MAX Transfection Reagent	ThermoFisher Scientific	13778150
Streptavidin magnetic beads	Pierce/ThermoFisher Scientific	88816

Tris-borate-EDTA (TBE) buffer, 10X	National Diagnostics	EC-860
Triton X-100	MilliporeSigma	T8787
Xfect Transfection Reagent	Clontech Laboratories	631317

Table S3. Oligonucleotides

EMSA1 (61 nt)	FAM-GACGCTGCCGAATTCTACCAGTGCCTTGCTAGGACATCTTTGCCCACCTGCAGGTTCACCC
EMSA2 (61 nt) (full complement)	GGGTGAACCTGCAGGTGGGCAAAGATGTCCTAGCAAGGCACTGGTAGAATTCGGCAGCGTC
EMSA3 (25 nt)	GCTGTCTAGAGGATCCGACTATCGA
EMSA4 (25 nt)	TGGGTGAACCTGCAGGTGGGCAAAGA
EMSA5 (62 nt) (half complement)	ATCGATAGTCGGATCCTCTAGACAGCTCCATGTAGCAAGGCACTGGTAGAATTCGGCAGCGT
EMSA6 (25 nt)	FAM-TCGATAGTCGGATCCTCTAGACAGC
DNA pulldown (80 nt)	BIO-$(TTT)_{10}$ CTCCCTTCTTCTCCTCCCTCTCCCTTCCC $(TTT)_7$
DNA pulldown complement	$(AAA)_7$ GGGAAGGGAGAGGGAGGAGAAGAAGGGAG $(AAA)_{10}$
NMR 16-mer	GCTATGGAGAACGGTA
IRE RNA (28 nt)	UCCUGCUUCAACAGUGCUUGGACGGAAC

Table S4. NMR Spectrum Parameters

Spectrum	Bruker Pulse Sequence	Size of FID (TD)	Spectral Width in ppm (SW)	NS	Others
N-HSQC	trosyf3gpphsi19.2	F1-^{1}H 1024/ F2-^{15}N 128	F1-^{1}H 12.00/ F2-^{15}N 36.00	16	--
HNCA	hncagp3d	F1-^{1}H 2048/ F2-^{15}N 40/ F3-^{13}C 64	F1-^{1}H 15.94/ F2-^{15}N 35.00/ F3-^{13}C 30.00	16	--
HNCACB	hncacbgp3d	F1-^{1}H 2048/ F2-^{15}N 40/ F3-^{13}C 128	F1-^{1}H 14.0029/ F2-^{15}N 35.00/ F3-^{13}C 80.00	16	--
HNCOCA	hncocagp3d	F1-^{1}H 2048/ F2-^{15}N 40/ F3-^{13}C 128	F1-^{1}H 15.94/ F2-^{15}N 35.00/ F3-^{13}C 30.00	16	--
HNCACO	hncacogp3d	TD: F1-^{1}H 2048/ F2-^{15}N 40/ F3-^{13}C 128	F1-^{1}H 14.0029/ F2-^{15}N 35.00/ F3-^{13}C 14.00	16	--
HNCOCACB	hncocacbgp3d	F1-^{1}H 2048/ F2-^{15}N 40/ F3-^{13}C 128	F1-^{1}H 15.94/ F2-^{15}N 35.00/ F3-^{13}C 80.00	16	--
HNCO	hncogpwg3d	F1-^{1}H 2048/ F2-^{15}N 40/ F3-^{13}C 128	F1-^{1}H 14.00/ F2-^{15}N 35.00/ F3-^{13}C 14.00	32	--
C-HSQC	hsqcetgp	F1-^{1}H 1024/ F2-^{13}C 256	F1-^{1}H 15.94/ F2-^{13}C 165.0	16	--
N-TOCSY	dipsihsqcf3gpsi3d	F1-^{1}H 2048/ F2-^{15}N 40/ F3-^{1}H 64	F1-^{1}H 15.94/ F2-^{15}N 35.00/ F3-^{1}H 15.94	16	--
CCCONH	ccconhgp3d	F1-^{1}H 2048/ F2-^{15}N 40/ F3-13C 128	F1-^{1}H 14.00/ F2-^{15}N 35.00/ F3-^{13}C 80.00	16	--
C-NOESY	noesyhsqcetgp3d	F1-^{1}H 2048/ F2-^{15}N 40/ F3-^{1}H 128	F1-^{1}H 14.00/ F2-^{13}C 75.00/ F3-^{1}H 14.00	32	Mixing Time (D8): 0.2 sec
N-NOESY	noesyhsqcetf3gp3d	F1-^{1}H 2048/ F2-^{15}N 40/ F3-^{1}H 128	F1-^{1}H 14.00/ F2-^{15}N 36.00/ F3-^{1}H 14.00	32	Mixing Time (D8): 0.2 sec
IPAP	hsqcetf3gpiaphsisp	F1-^{1}H 1024/ F2-^{15}N 600	F1-^{1}H 12.46/ F2-^{15}N 34.00	120	--
ssNOE	hsqcfpf3gpphwg	F1-^{1}H 2048/ F2-^{15}N 512	F1-^{1}H 13.02/ F2-^{15}N 34.00	32	Recovery Delay (D7): 7 sec

F1, 2, 3 – Nucleus are the channels and its corresponding nucleus.

Supplementary Figure S1

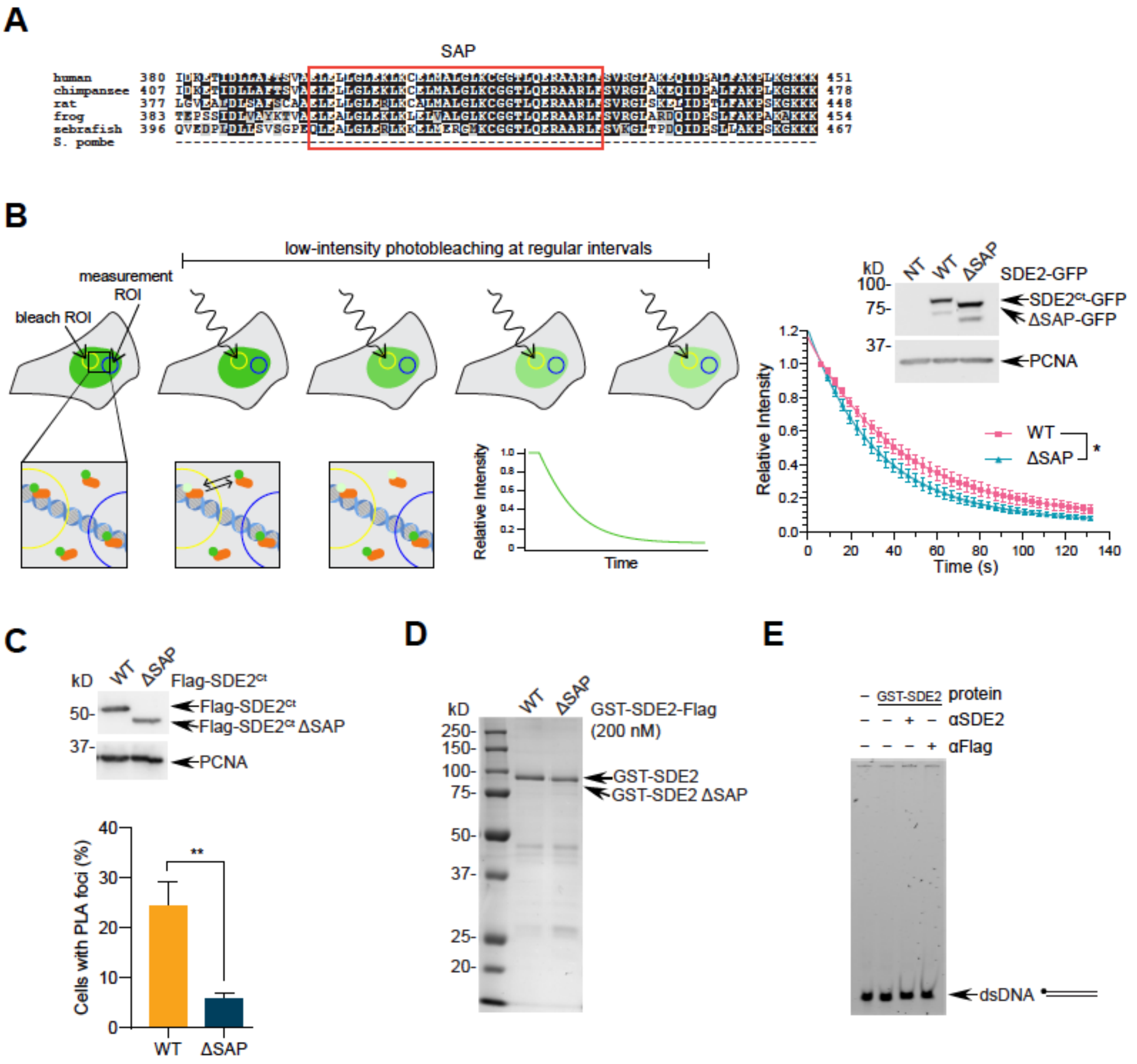

Supplementary Figure S1. Characterization of DNA binding by SDE2^{SAP}

(A) The sequence alignment of the SDE2 SAP domain from multiple species. The core SAP domain is marked with a red box. **(B)** Left: schematic showing how fluorescence loss in photobleaching (FLIP) is used to compare movement rates of nuclear proteins. Fluorophore-tagged proteins in the bleach region-of-interest (ROI) are partially photobleached and are allowed to exchange with non-photobleached proteins outside of the ROI, either through active movement or diffusion, before being bleached again. Bleaching steps are repeated at regular intervals until fluorescence signal is abolished or no longer decreases. Right: U2OS cells transiently expressing either SDE2-GFP WT or ΔSAP (Δ395-427) were subjected to FLIP while capturing images approximately every 3.5 s; curve fit to two-phase decay model, error bar: SD, *p<0.05, two-tailed t test. Western blots show proteins used for FLIP

experiments. **(C)** Top: Western blot showing proteins used for SIRF experiments. U2OS cells were transfected with either Flag-SDE2^{Ct} WT or ΔSAP and analyzed via Western blotting. Bottom: Percentage of replicating cells (positive for EdU-EdU signal) that contain PLA foci. Quantification of three independent experiments; Error bar: SEM, **p<0.01, two-tailed Mann-Whitney test. **(D)** Coomassie Blue staining of the purified GST-SDE2-Flag WT and ΔSAP used for EMSAs. **(E)** Control EMSA experiment for supershift. Purified GST-SDE2-Flag were incubated with 60-mer dsDNA, additionally incubated with antibodies at a 1:3 ratio where indicated. DNA: 50 nM, protein: 200 nM. Lane 1: no protein control; lane 2: GST-SDE2-Flag; lane 3: GST-SDE2-Flag + anti-SDE2; lane 4: GST-SDE2-Flag + anti-Flag. Neither dsDNA binding nor supershift was observed.

Supplementary Figure S2

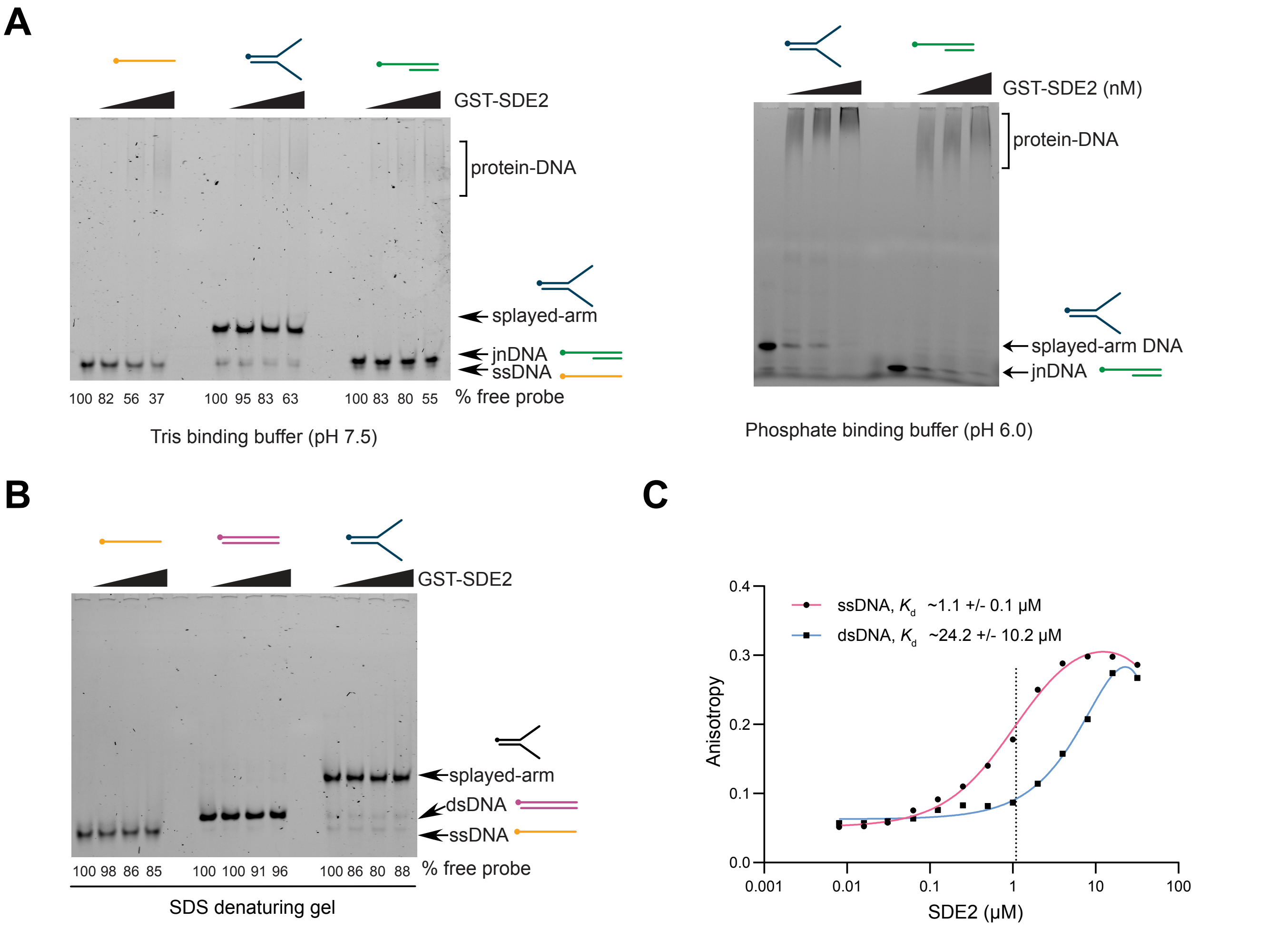

Supplementary Figure S2. ssDNA-specific DNA binding of SDE2^{SAP}

(A) EMSA of purified GST-SDE2-Flag incubated with 60-mer ssDNA, saDNA, or jnDNA (junction DNA). DNA: 50 nM, protein: 0, 50, 100, 200 nM. Note that EMSAs were performed in both Tris binding (pH 7.5) and phosphate binding (pH 6.0) buffers, showing that results are reproducible in independent buffers. **(B)** EMSA of purified GST-SDE2-Flag incubated with 60-mer ssDNA, dsDNA, or saDNA then run on a *denaturing* gel. DNA: 50 nM, protein: 0, 50, 100, 200 nM. **(C)** Increasing amounts of full-length GST-SDE2-Flag were incubated with FAM-labeled 60-mer ssDNA vs. dsDNA and analyzed using fluorescence anisotropy. Full-length SDE2 binds ssDNA with an apparent dissociation constant (K_d) of approximately 1.1 µM, but to dsDNA with a K_d of approximately 24 µM. Data fit to a one-site, total binding saturation curve. Dissociation constants are approximate due to challenges in reaching >90% purity for GST-SDE2-Flag at the scale required, due to its propensity to cleave in the large flexible region that makes up the bulk of the primary sequence.

Supplementary Figure S3

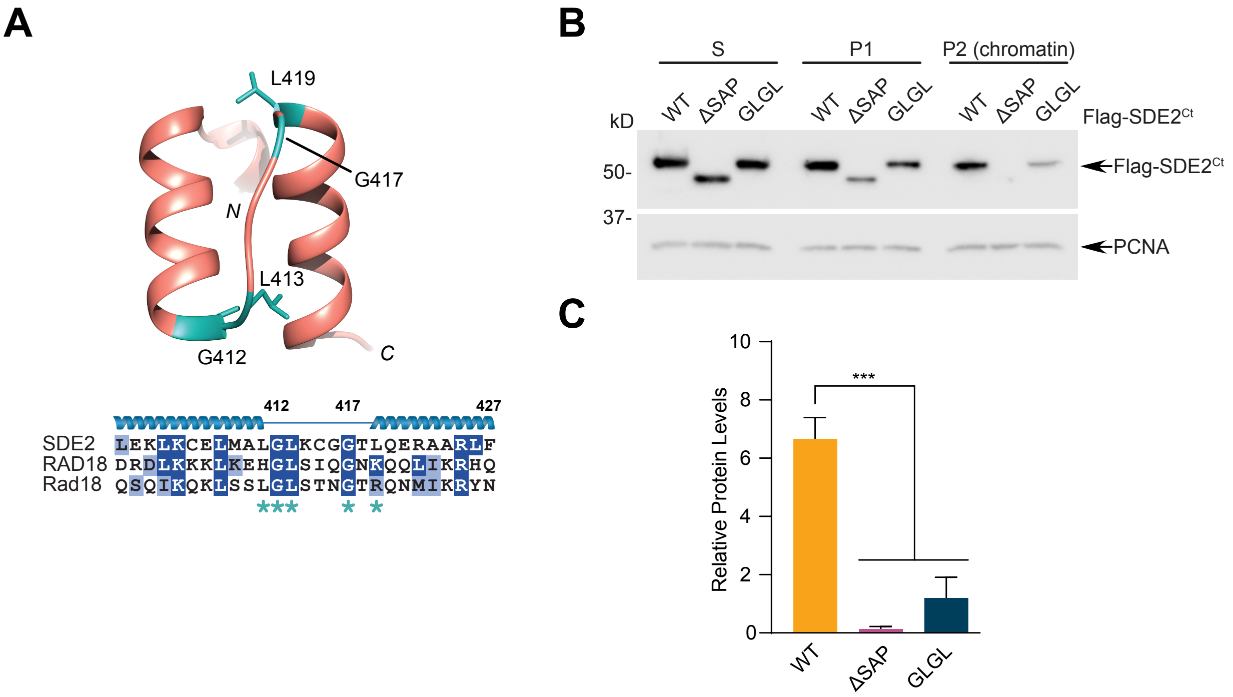

Supplementary Figure S3. Conserved loop-region residues are important for DNA binding. (A) A predicted structure of $SDE2^{SAP}$ (aa. 395-427), generated by Phyre2 based on primary sequence identity to known structures, with the most contributions coming from PDBs 2KVU (myocardin-like protein 1), 1ZRJ (E1B-55kDa-associated protein 5), 2DO1 (Hcc-1), 1H1J & 2WQG (Tho1), and 2RNN (Siz1). Residues corresponding to $Rad18^{SAP}$ binding mutants are highlighted in teal. **(B)** U2OS cells were transfected with Flag-$SDE2^{Ct}$ WT, ΔSAP (Δ395-427), or G412A/L413E/G417A/L419E (GLGL), fractionated to separate cytosolic and chromatin-associated protein pools, and analyzed by Western blotting. **(C)** Quantification of (B) from three independent experiments; error bar: SEM, ***$p<0.001$, one-way ANOVA.

Supplementary Figure S4

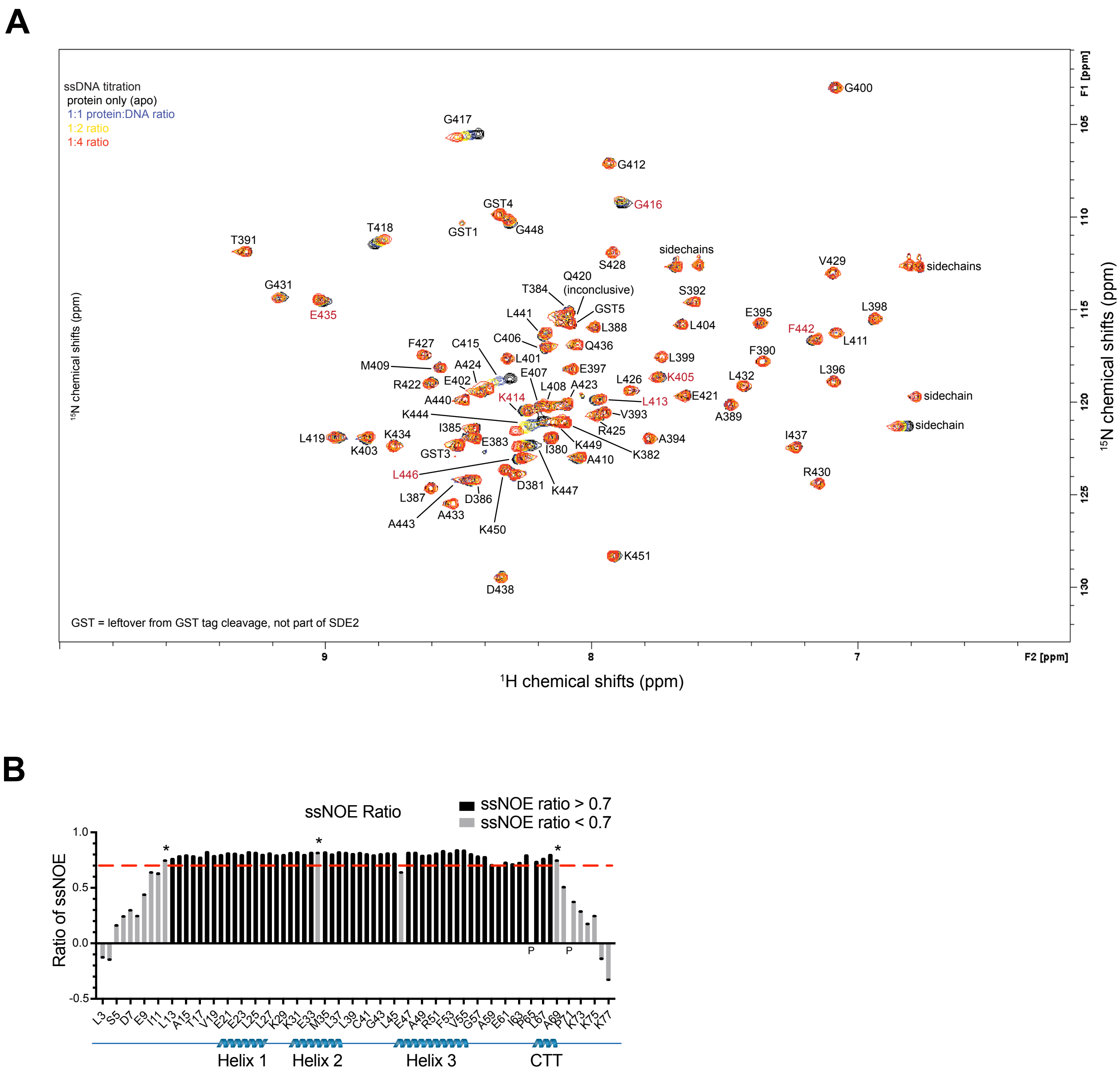

Supplementary Figure S4. HSQC analysis of SDE2^{SAP}

(A) Full assignment of ^{1}H-^{15}N HSQC spectra, showing all cross-peak assignments. Figure 3B was reused to show full assignment. Cross-peaks 1-5 are from residues left after the cleavage of the GST tag by the HRV 3C protease, and are not part of the SAP+CTT motif. **(B)** Backbone dynamics of apo SDE2$^{SAP+CTT}$. Plot of {^{1}H}-^{15}N steady-state NOE (ssNOE) ratio vs. residue number. A ratio of >0.7 (indicated by red dashed line) was used as the cutoff for selection of the TALOS (Torsion Angle Likelihood Obtained from Shift and sequence similarity)-predicted dihedral angle and structural calculation. P indicates prolines, which do not show up in {^{1}H}-^{15}N HSQC, while * indicates residues with strongly overlapping cross-peaks for which ssNOE values could not be accurately extracted.

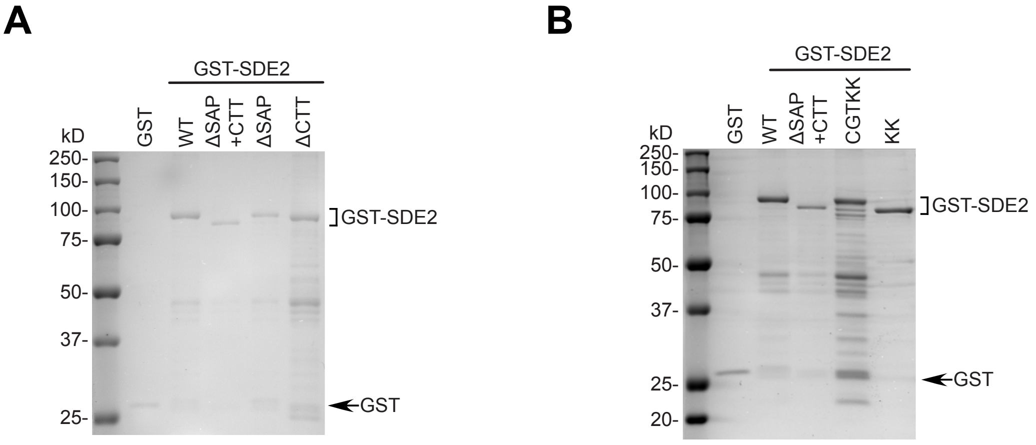

Supplementary Figure S5. Contribution of SDE2^{SAP} and SDE2^{CTT} for DNA binding
(A) Coomassie blue staining of purified GST-SDE2 variants used for EMSAs in Fig. 5D. **(B)** Coomassie blue staining of purified GST-SDE2 variants used for EMSAs in Figs. 5F and 5G.

Supplementary Figure S6

A

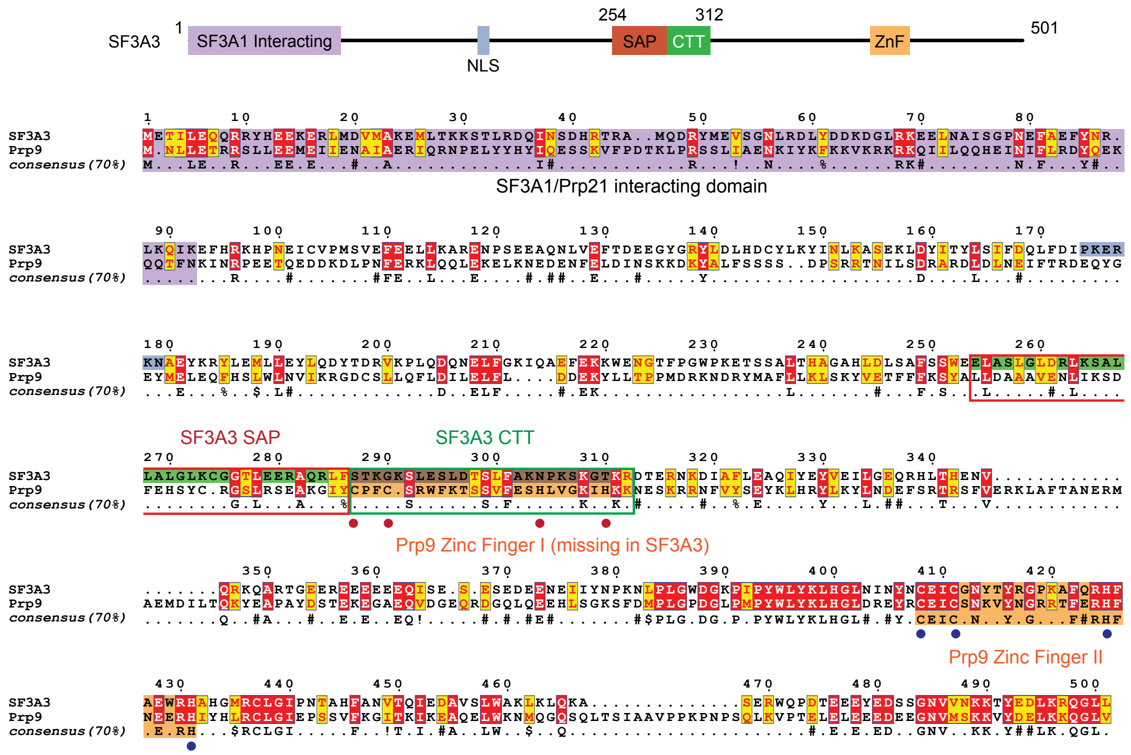

Supplementary Figure S6. Conservation of the extended SAP in SDE2 and SF3A3

(A) Sequence alignment of SF3A3 and its yeast homolog Prp9. The SF3A1/Prp21 interacting domain is colored purple, SF3A3 SAP and CTT are colored red and green, respectively, and the zinc fingers are colored orange. Notice that while SF3A3 and Prp9 both share the C-terminal ZnF motif that binds the spliceosome 15S intermediate, Prp9 contains an additional zinc finger at the location of the CTT in SF3A3. The positions of missing cysteine and histidine residues in the ZnF motif I of SF3A3 are marked with red dots.

Supplementary Figure S7

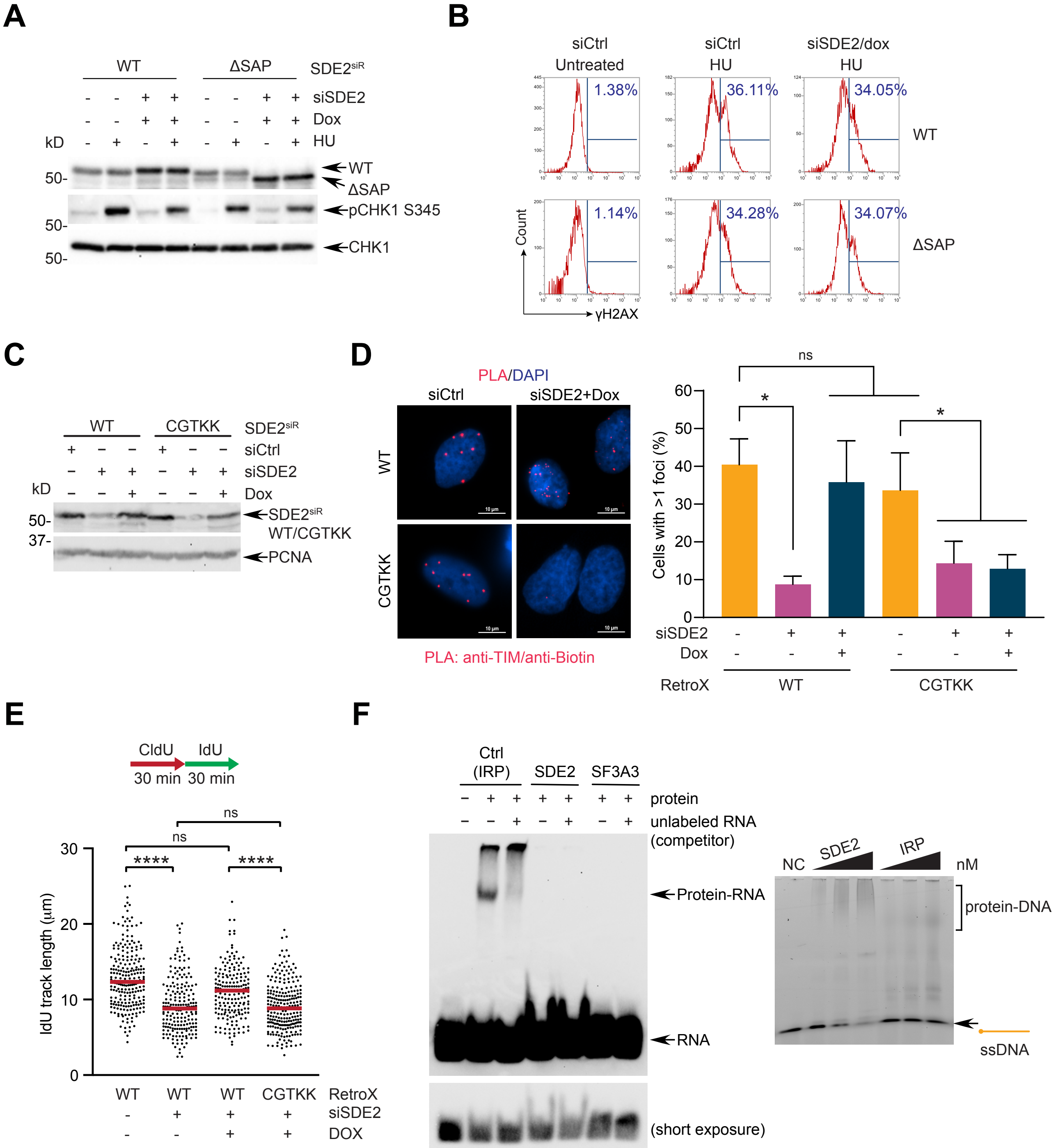

Supplementary Figure S7. Characterization of the SAP point mutants

(A) CHK1 phosphorylation at S345 after treatment of 2 mM HU for 4 h in Retro-X cells before after siRNA and dox induction to reconstitute SDE2 WT or ΔSAP. **(B)** Induction of γH2AX levels in in Retro-X cells treated with 2 mM HU for 4 h, measured by flow cytometry analysis of γH2AX Alexa Fluor 488. A representative result from two independent experiments is shown. **(C)** Western blotting that shows the complementation of SDE2 WT or SAP mutant (CGTKK; C415A/G417S/T418V/K444A/K447A) in Retro-X cells by siRNA transfection and Dox induction. **(D)** Left: Representative images of TIM:EdU PLA foci in the Retro-X SDE2 WT or SAP mutant cells following SDE2 siRNA transfection and dox induction. Scale bar: 10 μm. Right: quantification of cells positive for TIM-EdU PLA foci normalized to EdU-EdU PLA-positive replicating cells (>300 cells per condition, n=3, error=SEM, *p<0.05, 2-way ANOVA). **(E)** Dot plots of the DNA fiber IdU track length from Retro-X cells reconstituted with SDE2 WT or CGTKK point mutant (>200 tracks per condition, ****p<0.0001, ns, not significant, Mann-Whitney). **(F)** Left: competitive RNA electrophoretic mobility shift assay (REMSA) of control protein (iron-responsive protein: IRP), purified GST-SDE2, or GST-SF3A3 incubated with 28-mer 3' biotin-labeled IRE (iron-responsive element) RNA. Competitor is unlabeled IRE RNA and shows that binding is specific. biotin-RNA: 6.25 nM, unlabeled RNA 1 μM. Right: EMSA of GST-SDE2 or IRP incubated with 60-mer ssDNA, showing that IRP does not bind to ssDNA.
